# Increased Cardiovascular Mortality in Ecuador during COVID-19 Pandemic

**DOI:** 10.5334/aogh.4021

**Published:** 2023-04-05

**Authors:** Marco Fornasini, Ivan Sisa, Manuel E. Baldeon

**Affiliations:** 1 Facultad de Ciencias de la Salud y de la Vida, Universidad Internacional del Ecuador (UIDE), Quito, Ecuador; 2 Escuela de Medicina, Universidad San Francisco de Quito USFQ, Quito, Ecuador

**Keywords:** chronic noncommunicable diseases, COVID-19, Ecuador, mortality, cardiometabolic diseases

## Abstract

Before the COVID-19 pandemic, chronic noncommunicable diseases (NCDs), represented a high burden for low and middle-income countries. Patients with NCDs are at higher risk of COVID-19 and suffer worse clinical outcomes. We present mortality trends for myocardial infarction (AMI), stroke, hypertension (HT), and type-2 diabetes mellitus (T2DM) from 2005 to 2021 in Ecuador. The greatest increase in mortality observed in the pandemic was in AMI, T2DM, and HT. Factors related to COVID-19, health services, and patients with NCDs could contribute to these important increases in mortality.

## To the Editor

Chronic noncommunicable diseases (NCDs) are major health problems that exert a heavy burden in low- and middle-income countries (LMIC). Before the current COVID-19 pandemic, NCDs represented a high and increasing burden for the health care services in these countries [1]. LMIC have not been able to establish effective measures to prevent and manage the continuous growth of these pathologies. The burden of cardiometabolic diseases was exacerbated with the presence of the COVID-19 pandemic, as it unveiled the deficiencies to control them [1].

Ecuador, an LMIC, was severely affected at the onset of the pandemic with very high morbidity and mortality [2]. According to the National Institute of Census and Statistics of Ecuador (INEC), during 2020, there were 41,000 excessive deaths compared to 2019, and by November 2022, Ecuador officially reported 1.1 million COVID-19 cases and nearly 36,000 deaths, although excess mortality was around 90,000 [3, 4]. Ecuador has had a deficient registration and accuracy for COVID-19 diagnosis because it presents the lowest number of COVID-19 tests per million inhabitants in the region (170,173 per 1 million inhabitants) [5]. On the other hand, by 2021, vaccine coverage of the Ecuadorian population for COVID-19 was over 80%, which resulted in a decrease in excess mortality of 14% compared to 2020 [2].

Patients with COVID-19 commonly present a higher prevalence of cardiometabolic diseases than those without these conditions. In addition, these patients have a significantly higher risk of mortality compared to those without NCDs: diabetes (relative risk (RR): 1.5 to 3.0), hypertension (odds ratio (OR): 2.5 to 3.0), and cardiovascular disease (OR: 3 to 6.0) [6]. Also, COVID-19 infection has been associated with higher risk of adverse cardiometabolic outcomes. Thus, COVID-19 ambulatory patients have significantly higher risk of venous thromboembolism (VTE) (hazard ratio (HR): 2.74) and death (HR: 10.23) compared to noninfected individuals. Furthermore, hospitalized patients due to COVID-19 present significantly higher risk of VTE (HR: 27.6), heart failure (HR: 21.6), and stroke (HR: 17.5) [7].

**Figure 1:**
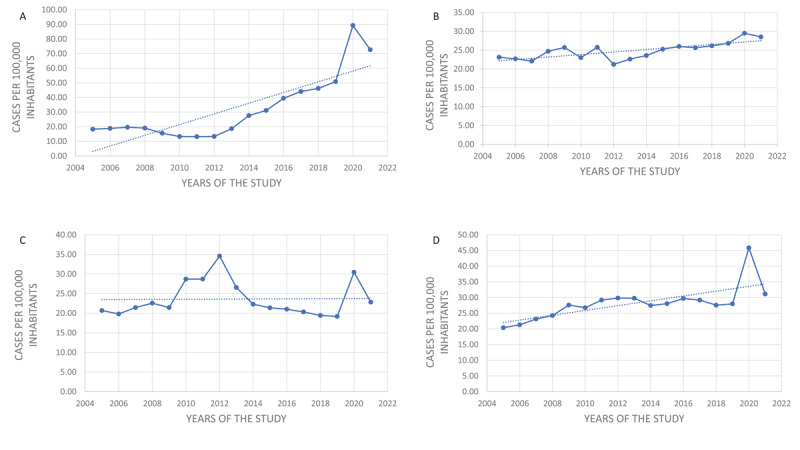
Mortality rate for chronic noncommunicable diseases in Ecuador, 2005–2021. Panel A—acute myocardial infarction; panel B—stroke; panel C—arterial hypertension; panel D—diabetes mellitus. Dotted lines are trend lines of mortality. Dashed lines represent trend lines for mortality through time.

Here we present NCDs mortality trends for Ecuador due to acute myocardial infarction (AMI, ICD-10: I20–I25), stroke (ICD-10: I60–I69), arterial hypertension (AHT, ICD10: I10–I15), and type 2 diabetes mellitus (T2DM; ICD-10: E10–E14) from 2005 to 2021. Crude mortality data were collected from INEC. Table 1 and Figure 1 show the crude mortality rate due to cardiometabolic diseases before and during the COVID-19 pandemic. The greatest increase in mortality was due to AMI, followed by T2DM, AHT, and stroke mortalities. The increase in mortality for cardiometabolic diseases in 2020 represented 30.4% (12,504) of the increased mortality for all causes (41,077). For 2021, although the rates of cardiovascular mortality are higher than those during the prepandemic period, these are lower than for 2020. It is likely that vaccination, increasing prevalence of infection, improvement of patient´s management, and implementation of general protecting measures could have contributed to this decrease in mortality. In Ecuador, vaccines became available in late 2020 and were highly effective to prevent and protect against severe infection and hospitalizations. Of notice, age-adjusted mortality rates for cardiometabolic diseases in Ecuador were slightly higher than crude ones during 2019–2021, considering the World Health Organization’s (WHO) world standard population. Furthermore, proportion mortality rates, excepting stroke, presented a minor increase in 2020 compared to 2019.

**Table 1:** Mean mortality rate and crude mortality rate for chronic noncommunicable diseases in Ecuador, 2005–2020.


	MORTALITY RATE MEAN ± SD/100 000 INHABITANTS 2005–2019	MORTALITY RATE PER 100 000 INHABITANTS 2020	MORTALITY RATE PER 100 000 2021	PERCENT OF CHANGEPRE-PANDEMIC VS 2020 (%)

**Acute myocardial infarction**	25.9 ± 13.1	89.3	72.7	243.6%

**Stroke**	24.3 ± 1.8	29.5	28.5	21.4%

**Arterial hypertension**	23.2 ± 4.4	30.4	22.8	48.3%

**Type 2 diabetes mellitus**	26.8 ± 3.1	45.8	31.1	70.9%


Several factors could have contributed to the important increases in NCDs mortality: (1) factors related with the pandemic (limited knowledge on the natural course of new SARSCoV2 infection, limited immunity, hyperinflammatory response [6], initial absence of treatments and vaccines, cultural limitations to implement general preventive measures); (2) factors related with health services (absence of trained personnel and means to treat infected patients, constrained budgets, use of clinical and emergency facilities for COVID-19 patients only, limited clinical facilities to treat patients with NCDs, limited follow-up of chronic patients); and (3) factors related with patients with NCDs (immunosuppression, fear to contamination in clinics, limited access to NCDs—specialists, treatments, and emergency services). It will be important to strengthen health care systems and to educate the population to control these risk factors to reduce the vulnerability of the population during health emergencies.

## Data accessibility statement

All authors had access to the data and a role in writing the manuscript.
